# Full-elevational gradient dataset on moth diversity and abundance in a temperate mountain range

**DOI:** 10.1038/s41597-026-06837-9

**Published:** 2026-02-12

**Authors:** Oldřich Čížek, Pavel Marhoul, Tomáš Kadlec, Oto Kaláb, Tomáš Jor, Antonín Hlaváček

**Affiliations:** 1 Hutur NGO, J. Purkyně, 1616, 50002 Hradec Králové, Czech Republic; 2 Beleco NGO, Na Zátorce 10, 16000 Prague, Czech Republic; 3 Department of Ecology, Faculty of Environmental Sciences, Czech University of Life Sciences Prague, Kamýcká 129, Prague, 16500 Czech Republic; 4 Department of Physical Geography and Geoecology, Faculty of Science, University of Ostrava, Chittussiho 10, 710 00 Ostrava, Czech Republic; 5 Department of Zoology, Faculty of Science, Charles University, Viničná 7, Prague 2, Prague, 12800 Czech Republic

**Keywords:** Entomology, Biodiversity

## Abstract

Climate change is reshaping ecosystems worldwide, yet our ability to quantify its long-term impact across taxa is limited by a lack of reliable and comparable data. Here, we present a systematically collected long-term dataset spanning nearly a decade (2012–2021), documenting the diversity, abundance, and distribution of 439 moth species (Lepidoptera: Heterocera) from the Czech part of the Giant Mountains, a region entirely protected as Krkonoše National Park. Using standardised light traps, we sampled 982 localities across an area of 550 km², yielding a total of 64,776 specimens. Localities are accompanied by *in-situ* assessments of vegetation characteristics and management regimes, complemented by topographical derivatives and ecosystem information retrieved post-hoc from open spatial data. The dataset provides a valuable resource for investigating spatial and temporal patterns in moth diversity and abundance, as well as for evaluating the effects of different management practices, supporting both basic and applied research.

## Background & Summary

In recent years, we are witness of a massive decline in insect abundance and diversity^1,2^, which, besides the effects of weather anomalies^3,4^, is driven mainly by human activity and habitat loss^5,6^. Among the most threatened habitats are mountain ranges, especially mountain grasslands and shrublands, which have undergone biome conversion exceeding 73%, while only 24.6% are currently protected^7,8^. Even though mountains, with their diverse biotope gradients, have traditionally been regarded as stable refuges capable of sustaining a wide range of species during periods of ecological stress^9,10^, a combination of ongoing habitat loss and unprecedented temperature rise may lead to the complete disappearance of specific habitats that harbour relict and narrowly adapted species^11–13^. Despite increasing attention to the impacts of climate change over the past two decades, we still lack reliable and comparable long-term data on its effects across most taxa, including nocturnal Lepidoptera.

Nocturnal Lepidoptera (formerly Heterocera), hereinafter called moths, represent a paraphyletic, hyperdiverse, functional group, encompassing approximately 75 – 85% of the 160,000 described Lepidoptera species^14,15^. A remarkable future of this group lies in the well-documented natural history of both adults and larvae, at least in the Palearctic region. With characterized adult phenology, habitat and microhabitat preferences, and larval host plants^16,17^, moths represent an ideal focal group for both basic research, such as studies on distribution^6^, population dynamics^18^, and macroecological patterns^19,20^, and applied research assessing habitat quality and degradation^21,22^, conservation and management practices^23,24^, or the effect of climate change on entire community assemblages^25^. While most of the aforementioned studies draw upon standardised sampling protocols and existing faunistic records, their scope is frequently constrained by the limited availability of such data.

Here, we present a long-term dataset on the diversity, abundance, and distribution of 439 moth species from the Czech part of the Giant Mountains, which is entirely protected as Krkonoše National Park (Czech Republic), systematically collected over nearly a decade (2012–2021) using standardised light traps. Our study encompasses 982 collection sites across the open habitats in the Krkonoše National Park, spanning an area of 550 km², and with a total of 64,776 collected specimens. Study sites are accompanied by *in-situ* assessment of vegetation characteristics and management regimes, and complemented by topographical derivatives and ecosystem information retrieved post-hoc from open spatial data.

## Methods

### Study area and research projects

**Fig. 1:**
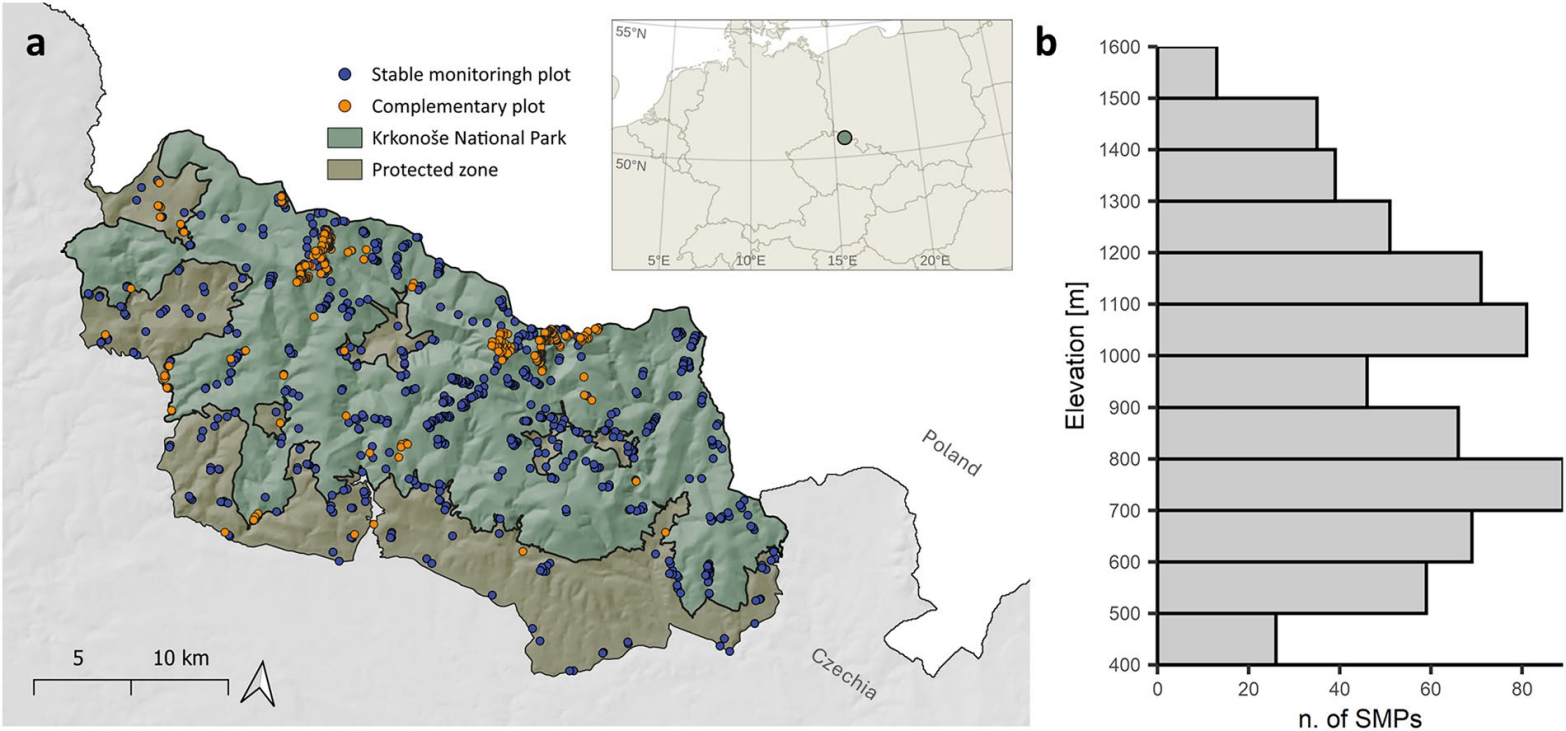
(**a**) Map of the study site indicating Krkonoše National Park and its Protected zone, with colour-coded monitoring plots (Stable monitoring plot (SMP) – blue; complementary plot – orange). (**b**) Histogram showing the distribution of SMPs along the elevation gradient.

The presented study took place in the Czech part of the Giant Mountains (also known as the Krkonoše /czech/ or Riesengebirge /german/) in the north Czech Republic, which are entirely protected as the Krkonoše National Park, hereafter referred to as the study area or Krkonoše National Park, see Fig. 1a). The Giant Mountains form part of the High Sudetes, originating during the Variscan orogeny^26^. They consist of two semi-separated ranges: the eastern range, with the highest peak Sněžka (1,604 m a.s.l., prominence 1,202 m), and the western range, with the highest peak Vysoké Kolo (1,509 m a.s.l., prominence 335 m). The elevation gradient of the study area, ranging from 400 to 1,600 m a.s.l., with the forest line averaging 1,230 m a.s.l.^27^, offers us a unique opportunity to investigate diverse habitat composition, ranging from rural temperate meadows and intensively managed pastures to alpine treeless biotopes and glacial cirques

Data were collected using the same methodology between 2012 and 2021, focusing on open and semi-open habitats within Krkonoše National Park. Our sampling design involved two types of research plots: a) Stable monitoring plots (SMPs), where data were collected during four sampling periods within a single year (late May, late June, late July, and late August or early September, see *meta.xlsx* table). Each SMP was visited only within that year and was not sampled across multiple years; b) Complementary plots (CPs), sampled once to three times, to cover a broad spectrum habitats not covered by SMPs and to capture the widest possible range of nocturnal moth species. A total of 645 SMPs and 337 CPs were systematically surveyed, see Fig. 1a. The distribution of SMPs was designed to include all major regions of both mountain ranges, from west to east and from the lowest (400 m a.s.l.) to the highest (1,600 m a.s.l.) elevations. The spatial pattern of SMPs reflected the distribution of treeless habitats and habitat diversity within each elevation. The smaller number of SMPs at the lowest altitudes (400–500 m a.s.l.) was attributed to higher anthropogenic pressure, the decrease in the number of SMPs between 800–1,000 m a.s.l. was attributed to a higher proportion of forest stands, and finally, the smaller number of the research plots at the highest altitudes (1,300–1,600 m a.s.l.) was a consequence of the overall smaller area, see Fig. 1b.

### Data collection and curation

We focused on moths from the following groups: Bombycoidea, Cossidae, Drepanoidea, Geometroidea, Hepialoidea, Lasiocampoidea, Limacodidae, and Noctuoidea. To assess the moth community, we placed at each monitoring point one automatic portable light trap^22,28,29^. Moths were attracted to the traps (70 cm in height) equipped with two luminous LED light strips (25 cm in length, 8 W, total luminous flux ~ 400 lm in wavelength of 400–420 nm; partly UV to blue spectrum, which is most efficient for moth attraction^28^ powered by 7.2 Ah/12 V lead batteries and euthanized by chloroform vapours (CHCl_3_), with the exception of conspicuous and easily identifiable moths (i.e., Sphingidae), which were only recorded and then released. Traps were exposed from dusk till dawn, under the most suitable weather conditions for moth trapping, avoiding heavy rain, strong wind, and full moon phases. Therefore, the intervals between sampling periods within a season could vary by several days. The samples were stored in paper bags and maintained at −22 °C prior to species identification. The nomenclature used in this study follows the checklist of Lepidoptera of the Czech Republic^30^. For each species, the conservation status was assigned according to the current Czech Red List of threatened species^31^. Moreover, 44 species were assigned to the indicator category, representing taxa with strong oreophytic habitat preferences and designated as focal species for the Giant Mountains.

All fieldwork was conducted in accordance with the applicable legislation of the Czech Republic and the European Union. Collecting and entry permits required for research within the National Park were obtained from the Krkonošský Národní Park Administration in Vrchlabí for all conducted projects. The presented study conforms to the legal requirements of the Czech Republic, where the study was conducted, as outlined in Act No. 114/1992 Coll. ‘Act of the Czech National Council on the Protection of Nature and Landscapes’. None of the captured nocturnal moths are subject to 48§ of Act No. 114/1992 Coll., on Nature and Landscape Protection, dealing with protected species of animals and plants, nor IUCN, CITES, or any other international conventions, except the willowherb hawkmoth (*Proserpinus proserpina* (Pallas, 1772)), which is protected under Annex IV of the Natura 2000 directive and also 48§ of Act No. 114/1992. A single individual was observed resting on the light trap at dawn in 2020; it was identified and released.

During each sampling period, vegetation structure and management type were assessed at each SMP, and mean values were then calculated for the entire sampling year. Vegetation structure was evaluated in 10 m buffer around sampling point, by recording minimum, mean, and maximum height (cm), proportion of sparse vegetation on semi- quantitative scale (1 – non to 5%, 2 –6% to 30%, 3 – more than 30%), number of blooming dicot species, and availability of nectar on semi-quantitative scale (0 to 10, in increments of 1). Management was assessed also within a 10 m buffer by recording the proportion of area under cattle, horse, or sheep grazing, mowing, or left unmanaged. All management categories summed to 100% of the assessed area.

### Spatial environmental data processing

In addition to *in-situ* assessment of site characteristics, available national open spatial datasets were used to obtain terrain and habitat characteristics of all sampling plots (both SMPs and CPs). All characteristics were calculated for a 30 m buffer around each sampling point, which is estimated to be the effective range of portable light traps^32^. All general processing of spatial data was handled in the R version 4.3.3^33^, using *lidR*^34,35^, *sf*^36,37^, and *terra*^38^ packages.

For terrain characteristics, we utilised the high-resolution laser scanning (LiDAR) digital terrain model (DTM) point cloud dataset “DMR5G”^39^. At each sampling site, point clouds were extracted within a 40 m radius. The extra 10 m beyond the 30 m data-extraction radius minimised edge artefacts during DTM interpolation. For each sampling site, we derived elevation, slope, aspect, modified Vector Ruggedness Measure (VRML), and Heat Load Index (HLI). Elevation data were represented by raw interpolated DTM raster values in meters. The slope and aspect were calculated in degrees using the *terrain* function from the *terra* package^38^, using the Horn algorithm^40^. The VRML, a unitless measure reflecting terrain heterogeneity, was calculated using the *vrm* function from the *spatialEco* package^41^, modified following Dilts *et al*.^42^. Heat Load Index, a measure of the potential solar radiation received by a surface, ranging from 0 (coolest) to 1 (hottest), considering aspect and slope was calculated with the *hli* function from the *spatialEco* package^41^, following McCune & Keon^43^. Each of the aforementioned characteristics is represented in the data by mean, standard deviation, and 1st and 99th percentiles (representing local minima and maxima), except the aspect, which is represented by the percentage of separate categories describing slope (flat, N, NE, E, SE, S, SW, W, NW).

The biotope type on each study plot was assessed using the “Consolidated Layer of Ecosystems” (CLES) framework^44,45^. This dataset synthesises various European sources (including land use, land cover, forestry, agriculture, etc.), providing complex information on the distribution of ecosystems in the focal area. Data, primarily at a scale of 1:10000^45^, are available as spatial polygon features delineating specific ecosystems. For each sampling point, we derived the proportion of each ecosystem, the number of patches, and the proportion of the largest patch (Largest Patch Index, LPI).

## Data Records

The dataset is publicly available on Figshare^46^. The data consist of two XLSX tables: one containing sampled species and their abundances (*species.xlsx*), and the other containing environmental variables that describe the sampling plot characteristics (*site_env.xlsx*). A metadata file (*meta.xlsx*) accompanies the dataset, providing information on the columns in the environmental data (*site_env.xlsx*) and on the sampling period for each study site. The decimal separator is a comma.

The *species.xlsx* table provides species-level data and observed abundances at each sampling site. It contains 18,133 rows (observations) and 10 columns: *site_id* – unique plot identifier; *family*, *genus*, and *species* – taxonomic information; *abundance*; *day*, *month*, and *year* of sampling; *redlist* – conservation status according to the Czech Red List of Invertebrates^31^; *bioindicator* – status according to Krkonošský National Park assessment^47^.

The *site_env.xlsx* file contains information about each sampling site. It includes 982 rows (sampling plots) and 72 columns. The first four columns contain general site information: *site_id –* unique plot identifier; *latitude*, *longitude* – coordinates; *type* of the sampling plot – SMP or CP. The rest of the columns are divided into four groups representing different environmental characteristics and are distinguished by prefixes:

- v_* – in-situ vegetation characteristics: *v_min*, *v_max, v_mean –* minimum, maximum, and mean vegetation height; *v_sparse –* relative area of sparse vegetation; *v_n_avail –* availability of nectar resources; *v*_*n_div –* number of flowering dicot species.
- m_* – in-situ management characteristics: *m_no*, *m_mow*, *m*_*cattle*, *m_horse*, *m_sheep –* relative area under different management types: unmanaged, mown, cattle-grazed, horse-grazed, and sheep-grazed.
- t_* – DTM derived topographic characteristics: t_elevation*, t_slope*, t_VRML*, t_HLI*, t_aspect*. The first four terrain characteristics were quantified as: *_mean, *_sd – standard deviation, *_p01 – 1st percentile (minimum), *_p99 – 99th percentile (maximum). Aspect is described in nine separate columns for each slope direction category: *_flat*_N, *_NE, *_E, *_SE, *_S, *_SW, *_W, *_NW.
- es_* – ecosystem characteristics derived from CLES: *es_np* – number of ecosystem patches; *es_LPI* – largest patch index. The remaining 30 columns represent relative ecosystem area, with detailed column names available in *meta.xlsx*.

The metadata file, *meta.xlsx*, includes concise descriptions of the column names in *site_env.xlsx*, as well as detailed records of the sampling dates for each of the SMPs and CPs. At several sampling points, we did not collect any specimens.

## Technical Validation

All authors and volunteers conducting fieldwork were instructed on light trap assemblage and placement by the corresponding author of this study. Moreover, the authors always supervised volunteers during trap placement to ensure consistency. Vegetation data were collected by the authors of this study, using the same semi-quantitative criteria and following prior calibration to ensure consistency. All specimens were identified by experts, Tomáš Kadlec and Michal Zapletal.

The *lidR* package^34,35^ was used to handle point cloud data and interpolate a 0.5 m resolution DTM using the local kriging algorithm implemented from the package *gstats*^48^. The number of points in the point cloud varies from 0.04 to 3.52 (0.6 ± 0.54), and the mean kriging error varies from 0 to 0.012 m (0.0002 ± 0.0008).

## Usage Notes

The dataset can be used for multiple research applications, including:

- Elevation gradient studies on the spatial distribution. The dataset allows examination of species turnover, environmental filtering, and community assembly along elevational gradients. Data on geometrid moths have already been published as part of a global meta-analysis^19^.
- Assessing species’ niche width in the context of range shifts driven by climate change by combining records across multiple habitats and years.
- Evaluating the impact of overall light pollution within a strictly protected area.
- Benchmark data for conservation studies to improve understanding of optimal management regimes at both species and community levels.
- Establishing baseline data on species phenology to support trait-based and natural history studies. The dataset provides detailed time-stamped records suitable for examining seasonal activity patterns.
- Contributing to national and European checklists and red lists of species by providing reliable occurrence and abundance data essential for conservation assessments.

## Data Availability

The dataset is publicly available on Figshare (10.6084/m9.figshare.30290536).

## Code availability

An R script containing the full spatial data processing workflow is available on GitHub repository (https://github.com/antoninhlavacek/Moth_Krkonose), with routines for data download, using *httr*^49^ and *xml2*^50^ packages, processing, and site-specific extraction.
